# Nutritional status of primary school children and their caregiver’s knowledge on malnutrition in rural and urban communities of Ekiti State, Southwest Nigeria

**DOI:** 10.1371/journal.pone.0303492

**Published:** 2024-05-13

**Authors:** Taofeek Adedayo Sanni, Olusegun Elijah Elegbede, Kayode Rasak Adewoye, Kabir Adekunle Durowade, Tope Michael Ipinnimo, Ayodele Kamal Alabi, John Olujide Ojo, Richard Dele Agbana, Mustapha Muhammad Raji, Oluseyi Adedeji Aderinwale, Mojoyinola Oyindamola Adeosun, Ademuyiwa Adetona, Opeyemi Oladipupo Abioye, Olumide Temitope Asake, Olanrewaju Kassim Olasehinde, Olawale Bashir-ud-deen Oni

**Affiliations:** 1 Department of Community Medicine, Federal Teaching Hospital, Ido-Ekiti, Ekiti State, Nigeria; 2 Department of Community Medicine, Afe Babalola University, Ado-Ekiti, Ekiti State, Nigeria; 3 Department of Ear, Nose and Throat, Federal Teaching Hospital, Ido-Ekiti, Ekiti State, Nigeria; 4 Department of Obstetrics and Gynaecology, Federal Medical Centre, Abeokuta, Ogun State, Nigeria; 5 Ogun State Hospital Management Board, Ministry of Health, Abeokuta, Ogun State, Nigeria; Innovative Aid, CANADA

## Abstract

**Background:**

Nutritional imbalance is an underlying cause of 2.6million death annually and a third of child’s death globally. This study assessed and compared the nutritional status of primary school children and their caregiver’s knowledge on malnutrition in rural and urban communities of Ekiti State.

**Methods:**

This is a cross-sectional comparative study carried out among 983 urban and rural primary school children in Ekiti State (495 in urban and 488 in rural) using interviewer-administered semi-structured questionnaire. A multi-stage sampling technique was used and data collected was analyzed using SPSS 23 with level of statistical significance set at p < 0.05.

**Results:**

Underweight and stunting were relatively higher in rural (6.5% and 22.7% respectively) than in urban (6.3% and 19.4% respectively) and these differences are not statistically significant (p = 0.898, p = 0.197). However, wasting, overweight and obesity were higher in urban (12.7%, 6.1% and 7.7% respectively) than rural (11.5%, 3.7% and 7.5% respectively) but the difference is not statistically significant. (p = 0.242). Majority of the caregivers in both settings had good knowledge of malnutrition though higher in urban mothers (89.5%) with statistical significance than their rural counterparts (71.5%). However, there is no significant association between caregiver’s knowledge and malnutrition in this study. Being in lower primary school class, relationship with caregiver, educational status of caregiver and occupation of caregiver were the common predictors of malnutrition among the school children in both community settings.

**Conclusion:**

Generally, the prevalence of malnutrition was high in both urban and rural primary school children in this study. However, while underweight and stunting were more prevalent among the children in the rural communities, wasting, overweight and obesity were more prevalent in the urban. The caregivers in both communities had good knowledge of malnutrition (better in the urban) but this is not good enough to bring a significant relationship with the occurrence of malnutrition in the children. Common predictors of malnutrition in both community settings are being in lower primary school class, relationship with caregiver, educational status of caregiver and occupation of caregiver. It is therefore recommended that regular continuous public enlightenment, nutritional education programmes and other programmes targeted at improving the economic power of the caregivers are measures that will improve the nutritional status of the primary school children.

## Introduction

Nutrition is a fundamental pillar of human’s health and development across the entire life cycle [1,2]. Nutrition is the intake of food to meet the body’s dietary need through different sources in order to enhance good nutritional status [2]. Nutritional Status is defined as the nutritional state of an individual, a population or a community [3,4]. Nutritional status is a balance between the intake of nutrients by an organism and the expenditure of these in the process of growth, reproduction and health maintenance. Therefore, children who are well-nourished with balanced diets are more likely to be healthy, productive and able to learn [5,6]. Nutrition and health status have powerful influence on a child’s learning ability and school performance [7]. Feeding practices in early life are important in cognition development of the child and the overall wellbeing of the child’s entire life [8–10]. Therefore, the life of a child is determined largely by the food given to cater for its rapid growth and development [3,11].

The developing countries are now witnessing Nutrition Transition; a term that describes shifts from the traditional dietary and energy expenditure patterns that significantly impact the nutritional status of a population [12,13]. During nutrition transition, the variety, quantity and price of available foods are altered, leading to changes in diet and lifestyle habits [12,14]. Young children from developing countries are increasingly making unhealthy food choices especially due to lack of knowledge and wrong perception towards healthy food. This is mainly because presently the concept of food has changed from a means of nourishment to a marker of lifestyle and source of pleasure [15–20].

Children who are overweight or obese are at higher risk of developing health problems such as type 2 diabetes, high blood pressure, respiratory problems and psychological problems of low self-esteem, depression and social isolation while under-nutrition causes increase susceptibility to infection, morbidity, reduced productivity, poor growth, low intelligence quotient behavioural problems etc [8,12,21].

Under-nutrition is still a major problem in sub-Saharan Africa with estimated 160million children being affected by stunting [21]. It has been estimated that 52% and 34% to 62% of school age children are stunted and underweight respectively [22–24]. In Nigeria, about 30% of the country’s school children have low Body Mass Index for age [25–27]. The prevalence of underweight and stunting in school children cohort study done in Lagos by National Centre for Health Statistics (NCHS) were 14.2% to 18.3% and 15.8% to 30% respectively [28]. In another study also done in Lagos, prevalence of overweight and obesity was put at 3.7% and 0.4% respectively [29,30]. Studies have documented prevalence rate of stunting, underweight and wasting as 27.7%. 29.9% and 25.5% respectively among school age children in Southeast Nigeria and 44.8%, 43.1% and 41.1% respectively among public primary students in Ibadan Southwest Nigeria [7,31].

School aged children attending one school or the other spend more time away from their parents and can thus develop instability of their dietary pattern. Also, influence from friends and media can affect the choice of food by mothers with subsequent effect on the formation and stability of pupils’ dietary practices and nutritional status [18]. Nutritional status of children is closely linked with multiples of factors and mother’s education is an important part [22,25,32–34]. Primary School pupils still fall under the dependent group of the society, with their feeding and nourishment mostly dependent on what is provided by their caregivers.

Mother’s knowledge affects child’s nutrition through her choices and health seeking skills related to nutrition, hygiene, preventive care and disease treatment. Mother’s responsibility to care for herself during pregnancy and her child through the most vulnerable stages of his/her life has significant effects on child nutritional status. Education helps mothers gain additional knowledge about the adequate intake of food for their children in terms of correct quantity, quality and frequency. Therefore the knowledge of caregivers on children nutrition would have significant effect on children’s nutritional status. A community based study which is an essential part of health monitoring at the community level is needed to assess and compare nutritional status among the rural and urban pupils in order to determine how much gap presently exists [3,35]. Knowing the nutritional status of the school children and the level of caregivers’ knowledge on malnutrition will assist in planning needed interventional programme [15–20].

## Materials and methods

This is a comparative cross sectional study carried out among selected primary school pupils and their caregivers in a rural and an urban Local Government Authority (LGA) in Ekiti State. Ekiti State in one of the six (6) states in the southwestern part of Nigeria. The state has 16 LGAs out of which four (4) are predominantly urban, four (4) are predominantly rural and others are rural-urban in nature. The state has a landmass of 5,887.89 km^2^. It enjoys a tropical climate, with two distinct seasons which are the rainy and the dry season. The vegetation is mainly guinea savannah in the north, while tropical forest exists in the south. It has an estimated total population of 2,384,212 (National Population Commission figures of 2006) with a 2021 projection of 3,816,784 based on an annual growth rate of 3.2%. [36]

A sample size of 1000 (500 in rural and 500 in urban) was calculated for this study using the minimum sample size formula for comparison of two proportions [37]; n = (Zα + Zβ)^2^ [P_1_(100-P_1)_ + P_2_(100-P_2_)] /(P1 –P_2_)^2^and then using the formulae 1+ (m-1) x ICC to compensate for the design effect (i.e cluster effect since cluster sampling was used at stage 4) [38].

A multistage sampling technique method was employed in the selection of the study participants. At stage 1, random sampling technique via balloting was used and Ado LGA and Ilejemeje LGA were selected as urban and rural LGA respectively. At stage 2, seven (7) wards were selected from lists of wards in each of the selected LGAs in stage 1 above via simple random sampling technique by balloting. At stage 3, two settlements each were selected using simple random sampling by balloting from each of the wards selected in stage 2 giving a total of fourteen (14) settlements in each LGA and questionnaires were allocated to each settlements using equal allocation. At the last stage, respondents were selected from each settlement by cluster sampling technique. The households were numbered and using the numbering sequence, eligible children and their caregivers were recruited until the allocated sample size was reached and in a household with more than one eligible child belonging to same caregiver, only one child was recruited via simple random sampling by balloting. All children currently attending primary schools (public or private primary schools) and their caregiver were included in the study, however, children with severe and life threatening diseases such as measles, complicated malaria, septicaemia, severe dehydration from acute or persistent diarrhoea etc., at the time of the study were excluded.

Data was generated using an interviewer administered questionnaire, weighing scale and standiometer for height measurement between 05/10/2019 and 20/11/2019. Research assistants were trained on application of the data collecting instruments and standard operating procedure (SOP) was itemized for the usage of the measuring instruments after necessary calibrations. Data collected were edited manually to detect omissions and to ensure uniform coding. The data was entered into a computer and analysis was done using Statistical Package for Social Sciences (SPSS) version 23 software package and WHO AnthroPlus version 1.0.4. Categorical variables were summarized as tables and proportions while continuous variables (e.g. age) were summarized as means (standard deviation) and values were compared between the urban and rural settlements. For questions that have the options of yes and no, all correct answers were scored one and incorrect answers zero. Chi-square test was used to determine associations among variables at bivariate level and statistical significance level was taken at p-value < 0.05 and confidence at 95%. Multivariate analysis was done to determine predictors of malnutrition using binary logistic regression analysis on variables that are statistically significant with malnutrition and ‘Enter Method’ was used to fit the model.

### Anthropometric indices

Anthropometric indices were calculated using the WHO AnthroPlus version 1.0.4 to obtain the nutritional status of school children. The values were compared with Z-score values of 2007 WHO Growth Reference Standards for 5–19 years [39] to determine children who are malnourished (stunted, wasted, undernourished, overweight or obese). The justification for use of 2007 growth reference standards is because it takes into consideration the growth pattern of children in developing countries. Children who were more than 2SD below the reference median (i.e. a Z-Score of less than -2) were considered to be undernourished i.e. to be stunted, wasted or to be underweight. Children with measurements between 2SD and 3SD (a Z-Score of -2 to -3) were considered to be severely undernourished. A BMI-for-age Z-score of ≤ +2SD was used to represent overweight and > +2SD represented obesity.

- Wasting—low weight for height with Z scores less than −2;
- Underweight—low weight for age with Z scores less than −2;
- Stunted—low height for age with Z scores less than −2.
- Overweight—high BMI for age with Z-score greater than +1 to +2
- Obesity—high BMI for age with Z-score greater than +2.
- 1 –Malnourished child = any child who is underweight, stunted, wasted, overweight or obese.
- 0 –Not malnourished child = any child who is not underweight, stunted, wasted, overweight or obese

Questions regarding caregivers knowledge were 20 with “Yes”, “No” and “I don’t know” responses. A correct response was assigned 1 point while an incorrect or I don’t know response was assigned 0. The total knowledge score varied between 0 and 20. A cumulative mean cut off was set at 10 such that respondents that scored above this value were deemed to have a good knowledge while those who scored 9 and below were regarded as having poor knowledge.

Ethical clearance for this study was obtained from the Health Research and Ethical Committees of the Federal Teaching Hospital, Ido Ekiti with protocol number ERC/2019/01/06/172A. Written informed consent was obtained from the caregivers and assent from the children in carrying out the study. Confidentiality was ensured through anonymous distribution of the questionnaire.

## Results

### Socio-demographic characteristics of primary school children in urban and rural settlements

The mean age of primary school children in urban community was 7.69 ± 1.95years with a range of 5-12years while that of the rural community was 7.66 ± 1.97years with a range of 5-12years. There was no significant difference in the mean age of the primary school children in both urban and rural communities (P = 0.791). In the urban community, more than half the students were in lower primary classes (Pry 1–3) while the majority of the children were in higher primary classes in rural (pry 4–5). Majority of the caregivers of the children in both communities were Mothers (78.8% in urban and 80.5% in rural). About 15.6% and 19.5% of primary school children in urban and rural communities respectively had their fathers as caregivers. However, in the urban community, 5.7% of the children had caregivers other than father. Majority of the caregivers in the urban community had tertiary education (52.7%) while the majority of the caregivers in rural had secondary education (48.0%). There existed a significant difference in the caregivers highest level of education at the public and private settings (P = <0.001). In the urban community, the occupation of majority of the caregivers was civil service while majority of caregivers in the rural community were traders. There existed significant difference in the employment status of caregivers in public and private primary school children (P = <0.001)–(**Table 1).**

**Table 1:** Socio-demographic characteristics of primary school children in urban and rural settlements.

Variable	Urban n = 495 Freq.(%)	Rural n = 488 Freq.(%)	Total n = 983 Freq.(%)	χ^2^	*P*-value
**Age (years)**					
5–7 8–10	207(41.8) 227(45.9)	185(37.9) 224(45.9)	392(39.9) 451(45.9)	1.826	0.281
≥ 10	61(12.3)	79(16.2)	140(14.2)		
Mean ± SD	7.69 ± 1.95	7.66 ± 1.97		0.264^t^	0.791
Range	5–12	5–12			
**Sex**					
Male	239(48.3)	262(53.7)	501(51.0)	2.873	0.090
Female	256(51.7)	226(46.3)	482(49.0)		
**Class**					
Pry 1	126(25.5)	70(14.3)	196(19.9)	25.128	**<0.001**
Pry 2	68(13.7)	78(16.0)	146(14.9)		
Pry 3	102(20.6)	89(18.2)	191(19.4)		
Pry 4	83(16.8)	98(20.1)	181(18.4)		
Pry 5	68(13.7)	79(16.2)	147(15.0)		
Pry 6	48(9.7)	74(15.2)	122(12.4)		
**Relationship of caregiver**					
Father	77(15.6)	95(19.5)	172(17.5)	29.847	**<0.001**
Mother	390(78.8)	393(80.5)	783(79.7)		
Others (Grand Ma, Sisters,Guardian etc)	28(5.7)	0(0.0)	28(2.8)		
**Caregiver’s highest educational level**					
None	2(0.4)	3(0.6)	5(0.5)	17.329^F^	**<0.001**
Primary	58(11.7)	49(10.0)	107(10.9)		
Secondary	174(35.2)	234(48.0)	408(41.5)		
Tertiary	261(52.7)	202(41.4)	463(47.1)		
**Caregiver’s employment status**					
Trading	204(41.2)	153(31.4)	357(36.3)	29.179	**<0.001**
Civil servant	151(30.5)	137(28.1)	288(29.3)		
Artisan	81(16.4)	120(24.6)	201(20.4)		
Farming	32(6.5)	48(9.8)	80(8.1)		
Unemployed	17(3.4)	29(5.9)	46(4.7)		
Others	10(2.0)	1(0.2)	11(1.1)		

P-values based on Independent Samples T test (NB: Overall mean age 7.68 ± 1.96).

### Nutritional status of primary school children in urban and rural communities

In the urban community, 6.3% of the primary school children were underweight and this value was slightly lower than the 6.5% of primary school children in the rural community who were underweight. However, this difference though slightly worse in the rural was not statistically significant (p = 0.898). Also, about one-fifth of the primary school children in this study were stunted, however, this was worse among the primary school children in the rural community (22.7%) than those in urban communities (19.4%). The difference in stunting between the urban and rural communities was however not significant (p = 0.197). This study also found that 12.7% of the primary school children in the urban community were wasted as against 11.5% of their counterpart in the rural community who were wasted. This shows that wasting is slightly higher in the urban communities than in the rural communities, however, this difference is not also statistically significant (p– 0.242). Furthermore, among the primary school children in the urban communities 6.1% were overweight and 7.7% were obese. These values in urban were higher than among the primary school children in rural communities where 3.7% were detected to be overweight and 7.5% were obese. These differences were however not statistically significant (p = 0.242)–(**Table 2).**

**Table 2:** Nutritional status of primary school children in urban and rural communities.

Nutritional status	Urban Freq.(%)	Rural Freq.(%)	Total Freq.(%)	Chi-square	p-value
**WAZ**					
<-2SD WAZ (Underweight)	28 (6.3)	29 (6.5)	57 (6.4)	0.016	0.898
≥-2SD WAZ	417 (93.7)	417 (93.5)	834 (93.6)		
**HAZ**					
<-2SD HAZ (Stunted)	96 (19.4)	111 (22.7)	207 (21.1)	1.661	0.197
≥-2SD HAZ	399 (80.6)	377 (77.3)	776 (78.9)		
**WHZ**					
<-2SD WHZ (Wasting)	63 (12.7)	56 (11.5)	119 (12.1)	4.186	0.242
-2SD–+2SD WHZ (Normal)	364 (73.5)	377 (77.3)	741 (75.4)		
>+2SD—+<3SD(Overweight)	30 (6.1)	18 (3.7)	47 (4.9)		
>+3SD (Obese)	38 (7.7)	37 (7.5)	76 (7.6)		

### Awareness and source of awareness of malnutrition among caregivers of children in urban and rural communities

Majority of caregivers in both urban and rural communities (83.8% and 76.8% respectively) had heard about malnutrition before. Sources of information for the urban community were majorly from nurses (28%), radio (21%) and television (19.8%). In the rural the major sources of information were community health extension workers (43.2%), nurses (31.7%) and radio (10.7%). There was significant difference in the level of awareness and the sources in urban and rural communities primary school children (P = 0.006 and <0.001 respectively)–**(Table 3).**

**Table 3:** Awareness and source of awareness of malnutrition among caregivers of children in urban and rural communities.

Variable	Urban n = 495 Freq.(%)	Rural n = 488 Freq.(%)	Total n = 983 Freq.(%)	χ^2^	*P*-value
**Ever heard about malnutrition**					
Yes	415(83.8)	375(76.8)	790(80.4)	7.618	**0.006**
No	80(16.2)	113(23.2)	193(19.6)		
Major sources of information**	**n = 415**	**n = 375**			
Doctors	37(8.9)	16(4.3)	53(6.7)	139.331	**<0.001**
Nurses	116(28.0)	119(31.7)	235(29.7)		
Community extension workers	47(11.3)	162(43.2)	209(26.5)		
Television	82(19.8)	19(5.1)	101(12.8)		
Radio	87(21.0)	40(10.7)	127(16.1)		
Internet/social media	42(10.1)	19(5.0)	61(7.7)		
Neighbour/family members	4(1.0)	0(0.0)	4(0.5)		

**—Multiple responses.

### Knowledge of caregivers on malnutrition in urban and rural communities

Majority of the caregivers in both communities knew the causes of malnutrition, how to detect it, some of the effect and measures in combatting it–**(Table 5).**

**Table 4:** Knowledge of caregivers on malnutrition in urban and rural communities**.

Variable	Urban n = 495(%)	Rural n = 488(%)	Total n = 983(%)	χ^2^	*P*-value
**Cause of malnutrition**					
Deficiency in intake of food / nutrients	437(88.3)	333(68.2)	770(78.3)	58.174	**<0.001**
Excessive intake of food and other nutrients	248(50.1)	287(58.8)	535(54.4)	7.517	**0.006**
Excessive consumption of junk/processed food	268(54.1)	270(55.3)	538(54.7)	0.140	0.709
Hereditary runs in the family	166(33.5)	140(28.7)	306(31.1)	2.693	0.101
**How to detect malnutrition**					
Regular weight check	446(90.1)	339(69.5)	785(79.9)	65.043	**<0.001**
Regular height check	280(56.6)	298(61.1)	578(58.8)	2.054	0.152
Regular eye check	223(45.1)	183(37.5)	406(41.3)	5.779	**0.016**
Regular temperature check	192(38.8)	160(32.8)	352(35.8)	3.850	0.050
**Effect of malnutrition**					
Poor child growth and development	447(90.3)	339(69.5)	786(80.0)	66.575	**<0.001**
Learning disability and poor school performance	425(85.9)	305(62.5)	730(74.3)	70.150	**<0.001**
Ridicule and poor relationship with friends	402(81.2)	278(57.0)	680(69.2)	67.744	**<0.001**
Predisposition to diabetes, hypertension and other chronic diseases	388(78.4)	232(47.5)	620(63.1)	100.367	**<0.001**
Predisposition to infections like diarrhea, measles, tuberculosis etc	393(79.4)	271(55.5)	664(67.5)	63.827	**<0.001**
**Measures to prevent malnutrition**					
Regular intake of balanced diet	461(93.1)	367(75.2)	828(84.2)	59.47	**<0.001**
Feeding children at least 3 times a day	461(93.1)	356(73.0)	817(83.1)	71.304	**<0.001**
Maintaining a garden at home	257(51.9)	390(79.9)	647(65.8)	85.628	**<0.001**
Public enlightenment on food and nutrition	323(65.3)	433(88.7)	756(76.9)	76.263	**<0.001**
Inclusion of nutritional education into the school curriculum	437(88.3)	314(64.3)	751(76.4)	78.099	**<0.001**
Provision of school feeding program by the government	302(61.0)	395(80.9)	697(70.9)	47.326	**<0.001**

**: Multiple responses.

There existed significant differences in the knowledge of caregivers living in urban and rural areas as regards causes, how to detect, effects and preventive measures of malnutrition.

Overall, more than four fifth (89.5%) of the caregivers in the urban had good knowledge and about three quarter (71.5%) in rural area also had good knowledge of malnutrition. More than a quarter (28.5%) had poor knowledge of malnutrition in the rural area compared with just a tenth (10.5%) in the urban. There was difference in the knowledge of caregivers of primary school children in urban and rural communities (better knowledge in the urban) which is significant at P = <0.001 **(Table 5).**

**Table 5:** Overall level of knowledge of caregivers on malnutrition in urban and rural communities.

Knowledge	Urban n = 495 (%)	Rural n = 488(%)	Total n = 983(%)	χ^2^	*P*-value
Good (≥ 50%)	443(89.5)	349(71.5)	792(80.6)	50.738	**<0.001**
Poor (< 50%)	52(10.5)	139(28.5)	191(19.4)		

P-values based on Chi-square.

### Association between knowledge and malnutrition among caregivers in rural and urban communities

Poor caregiver’s knowledge on malnutrition is statistically significantly associated with underweight among primary school children in the rural communities (p = 0.010) but not so among the students in the urban communities (p = 0.341). Poor caregivers knowledge is also significantly associated with stunting among primary school children in the urban communities (p = 0.093) but no so among the children in the rural community (0.294). However, caregiver’s knowledge is not statistically associated with wasting, overweight and obesity in both the urban and rural communities (p = 0.197, p = 0.187)- **(Table 6).**

**Table 6:** Association between knowledge and malnutrition among caregivers in rural and urban communities.

Variable	WAZ			HAZ			WHZ				
Nutrition Knowledge	<-2SD WAZ (Under weight)	≥-2SD WAZ	*p-value	<-2SD HAZ (Stunted)	≥-2SD HAZ	*p-value	<-2SD WHZ (Wasting)	-2SD–+2SD WHZ (Normal)	>+2SD WHZ-<3SD (Over weight)	>+3SD WHZ (Obese)	*p-value
**Rural**											
Good knowledge	15(51.7)	308(73.9)	0.010	75(67.6)	274(72.7)	0.294	35(62.5)	278(73.7)	10(58.8)	26(68.4)	0.197
Poor knowledge	14(48.3)	109(26.1)		36(32.4)	103(27.3)		21(37.5)	99(26.3)	7(41.2)	12(31.6)	
**Urban**											
Good knowledge	27(96.4)	373(89.4)	0.341^F^	81(84.4)	362(90.7)	0.093^F^	52(82.5)	329(90.4)	26(86.7)	36(94.7)	0.187^F^
Poor knowledge	1(3.6)	44(10.6)		15(15.6)	37(9.3)		11(17.5)	35(9.6)	4(13.3)	2(5.3)	

### Predictors of malnutrition among primary school children in urban community

Being in lower primary class (pry 1–4), relationship with caregiver (father), educational status of caregiver (primary and secondary education) and occupation of caregiver (trading, artisan, farming, unemployment) were predictors of malnutrition among primary school children in urban communities—**Table 7**.

**Table 7:** Predictors of malnutrition among primary school children in urban community.

Variable	B	*p* value	OR	95% CI	
				Lower	Upper
**Age**					
5–9	0.856	0.319	2.353	0.438	12.652
≥ 10 ^REF^			1		
**Class**					
Primary 1	-2.388	0.004	0.092	0.018	0.473
Primary 2	-3.650	0.000	0.026	0.004	0.157
Primary 3	-2.430	0.002	0.088	0.018	0.420
Primary 4	-2.129	0.003	0.119	0.029	0.495
Primary 5	-1.202	0.067	0.300	0.083	1.088
Primary 6 ^REF^					
**Relationship caregiver**					
Father	2.796	0.002	16.372	2.765	96.939
Mother					
Others ^REF^					
**Educational status (Caregiver)**					
None	1.865	0.310	6.455	0.176	236.951
Primary	1.936	0.029	6.934	1.216	39.532
Secondary	-1.007	0.027	0.365	0.149	0.893
Tertiary ^REF^					
**Occupation (caregiver)**					
Civil servant ^REF^					
Trading	1.143	0.004	3.136	1.434	6.858
Artisan	1.315	0.002	3.724	1.594	8.701
Farming	-1.283	0.092	.277	.062	1.231
Unemployed	1.624	0.041	5.074	1.072	24.023
Others	-19.305	0.999	.000	.000	.
**Birth order of child**					
1^st^	-1.447	0.346	0.235	0.012	4.774
2^nd^	-0.445	0.770	0.641	0.033	12.590
3^rd^	-1.045	0.501	0.352	0.017	7.400
4^th^	2.459	0.145	11.698	0.427	320.738
5^th REF^					
**Type of toilet facility**					
None					
Open defecation	-0.300	0.745	0.741	0.121	4.520
Water closet	-0.578	0.478	0.561	0.113	2.774
Pit latrine	-0.439	0.413	0.645	0.225	1.844

B: Coefficient of Binary Logistic regression; OR: Odds ratio; 95% CI: 95% Confidence Interval.

Hosmer-Lemeshow goodness of fit = 10.740 (p < 0.217), Cox and Snell R squared = 0.288.

### Predictors of malnutrition among primary school children in urban community

Age (5-9years), primary school class of the student (pry 2), relationship with caregivers (father), education status of caregiver (secondary), occupation of caregiver (artisan), household head income (₦18,000 - ₦30,000), household income and toilet facility (pit latrine) were predictors of malnutrition among the rural community primary school children– Table 8A and 8B.

**Table 8:** a: Predictors of Malnutrition among primary school children in rural communities. b: Predictors of Malnutrition among primary school children in Rural Community Contd.

Variable	B	*p* value	OR	95% CI	
				Lower	Upper
**Age**					
5–9	-2.807	0.017*	1.120	0.694	3.610
≥ 10 ^REF^			1		
**Class**					
Primary 1	1.645	0.597	5.183	0.012	9.637
Primary 2	8.090	0.009	1.608	0.492	7.266
Primary 3	0.722	0.831	2.059	0.003	15.547
Primary 4	3.083	0.320	1.832	0.050	5.202
Primary 5	3.041	0.352	2.930	0.035	6.423
Primary 6 ^REF^			1		
**Relationship caregiver**					
Father	1.874	<0.001*	6.512	2.355	18.005
Mother	-0.426	0.399	0.653	0.243	1.757
Others ^REF^			1		
**Educational status (caregiver)**					
None	25.491	0.999	1.787	.000	.
Primary	0.619	0.545	1.857	.250	13.780
Secondary	2.852	<0.001*	17.316	6.484	46.247
Tertiary ^REF^			1		
**Occupation (caregiver)**					
Civil servant			1		
Trading	-3.664	0.020	0.026	0.001	0.566
Artisan	5.642	<0.001*	8.972	5.080	27.307
Farming	3.241	0.163	2.561	0.270	7.810
Unemployed	5.688	0.027	5.293	1.883	16.267
Others	16.885	1.000	4.734	1.245	15.264
**Monthly income (household head)**					
< ₦18,000	-0.373	0.603	0.689	0.169	2.808
₦18,000 –₦30,000	-1.049	0.080	0.350	0.108	1.133
> ₦30000					1
**Variable**	**B**	***p* value**	**OR**	**95% CI**	
				**Lower**	**Upper**
**Number of children**					
1	-24.753	0.998	2.447	0.464	12.907
2	-24.396	0.998	2.698	0.625	11.651
3	-25.278	0.997	1.449	0.374	5.609
4	-23.243	0.998	2.205	0.546	8.908
> 4 ^REF^					
**Birth order of child**					
1^st^	6.344	0.999	1.463	0.145	14.741
2^nd^	5.230	0.998	2.553	0.268	24.345
3^rd^	3.291	0.997	2.390	0.249	22.923
4^th^	1.574	0.999	14.428	1.249	16.673
5^th REF^					1
**Source of drinking water**					
Tap water	1.802	0.243	6.060	0.294	12.858
Well water	1.990	0.188	7.316	0.379	41.396
Borehole	2.794	0.081	6.344	0.712	15.118
Rain water	1.934	0.233	6.916	0.289	15.668
Stream water					
**Type of toilet facility**					
None	1.955	0.364	7.061	3.104	9.598
Open defecation	2.141	0.383	8.506	4.070	19.407
Pit latrine	4.086	0.006*	5.527	3.239	10.035
Water closet			1		
**Knowledge**					
Good	-0.173	0.542	0.841	0.482	1.468
Poor					

B: Coefficient of Binary Logistic regression; OR: Odds ratio; 95% CI: 95% Confidence Interval.

Hosmer-Lemeshow goodness of fit = 9.247 (p < 0.322), Cox and Snell R squared = 0.486.

## Discussion

This study found that the prevalence of malnutrition (under-nutrition and over-nutrition) is still generally high in Ekiti State, However, under-nutrition is worse among the primary school children in the rural communities than the urban communities despite the state being a beneficiary of the ongoing Federal Government School Feeding Programme in public primary schools. This may be a reflection that the public primary schools in urban may be benefiting more from the programme than those in the rural communities.

The overall prevalence of underweight in this study was 6.4% (6.3% in urban and 6.5% in rural community). The magnitude of underweight is slightly higher in the rural setting than in the urban setting. This may be due to lower income households earning capability and lower caregiver’s knowledge in the rural than urban. This low prevalence is similar to findings in Abakaliki Metropolis of Ebonyi state and Fayoum Governorate in Egypt where prevalence of 4.5% and 3.4% respectively were recorded [22,40]. It is lower than findings among Nigerian School children in a semi-urban area of Ogun state where underweight prevalence of 25.5% was reported and study done among rural and urban Nigerian School children where prevalence rate of 61.1% was documented for underweight [3,30]. However, the prevalence of underweight in this study is slightly higher in rural communities (6.5%) than in the urban community (6.3%). Higher prevalence of underweight in rural communities than urban were observed in study done among primary school children in urban and rural communities of Lagos State Nigeria, Anambra State Nigeria and Rajasthan India where underweight prevalence reported in rural settings (49.6%, 18.8% and 20.56% respectively) are higher than the urban rates (15.1%, 2.7% and 18.89% respectively) [41–43].

This study showed that the overall prevalence of stunting among primary school children in Ekiti state was 21.1% though higher in the rural (22.7%) than the urban community (19.4%). The burden of stunting in the rural primary school children is also slightly higher than the urban school children. This may be because of poor access to good nutritional diets, inadequate and inappropriate knowledge of caregivers as regard nutrition and/or poor access and utilization of the school feeding programme in the rural communities. This overall prevalence found in this study is higher than findings in a semi-urban area of Ogun state and in Western Nepal where overall prevalence rate of 14.2% and 13% were reported [30,44]. This study overall prevalence rate of stunting is also higher that of Harare with stunting prevalence of 7.4% but lower than it was documented in Ife Central of Osun State Nigeria, Ohaji Egbeme LGA of Imo State Nigeria, Fayoum in Egypt, Azad Nagar area of Bangalore and Burkina Faso where prevalence of 27.3%, 40.14%, 34.2%, 40.4% and 29.4% were reported [1,3,5,22,33,45]. The higher prevalence of stunting among school children in rural than urban found by this study is in keeping with findings Ife Central of Osun State (35.5% in rural, 18.8% in urban), Obafemi Owode LGA of Ogun State (46.2% in rural, 33.8% in urban), Lagos Nigeria (50.85% in rural, 16.6% in urban) and Anambra State Nigeria (3.3% in rural, 0.5% in urban) [3,33,42,43].

For wasting, this study found an overall prevalence of 12.1%; slightly higher in urban (12.7%) than rural (11.5%). These findings are similar to reports in Burkina Faso, Nepal and Karnakata India where prevalence of 11.2%, 12% and 13% respectively were documented [44–46]. The finding is higher than as documented for primary school children in Fayoum Egypt and Harare where prevalence of 0.9% and 4.1% were reported [1,7]. However, the finding is lower than as reported in Ife Central of Osun State and Obafemi Owode LGA of Ogun with prevalence reports of 16.8% and 22.2% respectively [3,30]. The slight difference in the prevalence of wasting with worse outcome in urban than rural as documented in this study is not in keeping with studies in Lagos Nigeria (24.3% rural to 13.65 urban) and Ouagadougou Burkina Faso (9.4% rural to 1% urban) where wasting in worse in rural than urban [43,47].

The overall prevalence of overweight found by this study was 4.9% among primary school children in Ekiti State with a higher value of 6.1% in urban than rural (3.7%). This higher overweight prevalence in urban than rural is in line with findings in Lagos Nigeria (15.1% urban, 13.2% rural), Anambra Nigeria (6.5% urban, 1.7% rural) and Rajasthan India (1.11% urban, 0.28% rural) [41–43]. The overall prevalence rate of 4.9% in this study is lower to an earlier study done in Ekiti State with overweight prevalence of 13.8% [48]. It is also lower than another Ekiti State study where prevalence of 37% and in Kaduna where 24.6% were documented. However, the finding is slightly higher than as found in Ebonyi State Nigeria, Ogun State Nigeria and Plateau Central/Centre-Ouest Regions of Burkina Faso with 1.2%, 3.0% and 2.0% prevalence [30,40,45].

This study also showed that the prevalence of obesity among primary school children in Ekiti State was 7.6%, though slightly higher in the urban (7.7%) than rural (7.5%). This may be due to change in dietary pattern of many homes away from the usual rich cultural diet to the less nutritious but enticing western diet. These findings in both community is close to the 5.2% reported among Dar s salaam primary school children [34]. The overall prevalence of obesity in this study is higher than as reported in Obafemi Owode LGA of Ogun State Nigeria (0.5%) and another done in public and private young school children Ekiti State (0.5%) but however lower than as reported in Egypt with 14.9% [22,30,48].

This study found that majority of the caregivers had good knowledge of malnutrition (89.5% in urban and 71.5% in rural). The knowledge demonstrated in urban was however much higher than in rural. Sources of information for urban caregivers were through nurses (28%), radio (21%) and television (19.8%) while most caregivers in the rural community got their information from Community Health Extension Workers (CHEW) and nurses. The high knowledge recorded in the rural community showed the good work of the CHEWs and nurses in the area and this is important in the fight against malnutrition. This finding is similar the study done in Nairobi Kenya where more than two thirds of the pupils have good knowledge of malnutrition.^19^ This study found that good knowledge of malnutrition and nutritional status by caregiver is associated with good nutritional status and poor knowledge is associated with malnutrition. This is similar to reports from studies done in Lagos Nigeria, Calabar South LGA Cross River Nigeria, Pancoran District of Southern Jakarta, Sulawesi Province of Indonesia and in Rwanda where good maternal knowledge is positively related to good nutritional status and vice versa [11,49–52].

It was also found that there was no significant association between knowledge of caregivers and malnutrition among the primary school pupils in Ekiti State. Though knowledge on malnutrition was high, the prevalence of malnutrition was also high in both urban and rural primary school children. This may be because either the caregivers were not applying their knowledge or because of low economic power of the caregivers, making it difficult to provide nutritious meals despite having good knowledge. This finding is similar to a study done in Sekhukhune District in South Africa and in Boyaca in Colombia, where no association was found among caregivers knowledge and nutritional status of children [53,54]. It is also similar to studies done in a district in the Western-North region of Ghana and Sokoto Metropolis, North-West Nigeria where it was documented that there was no significant association between mother/caregiver’s nutrition knowledge and child malnutrition [55,56].

This study found that some predictors of malnutrition among children attending primary schools in rural community of Ekiti State lower class in school (1–4), relationship with caregiver, educational status of caregiver and occupation of caregiver. However, in the urban community, predictors of malnutrition among primary schools children are age, being in lower primary school class, relationship with caregiver, educational status of caregiver, occupation of caregiver and household head income. Being in lower primary school class, relationship with caregiver, educational status of caregiver and occupation of caregiver are predictors common in both settings. The general findings in this study is similar to findings to studies in Fayoum Egypt, Burkina Faso and Owerri Nigeria, where predictors of malnutrition are age, household occupation, maternal educational status, dietary nutrition, family size and income of caregivers [4,17,42].

## Conclusion

The prevalence of undernutrition (underweight, stunting and wasting) is still high in Ekiti state. While underweight and stunting were higher among primary school children in the rural communities, wasting was higher among primary school children in the urban communities. However, prevalence of overnutrition (obesity and overweight) was higher in urban community primary school children than their rural community counterparts. The majority of primary school children caregivers in both urban and rural communities had good knowledge of malnutrition (though better in urban than in the rural communities) especially as regards causes, detection and prevention. However, there is no significant association between caregiver’s knowledge and malnutrition in this study. Common predictors of malnutrition in both community settings are being in lower primary school class, relationship with caregiver, educational status of caregiver and occupation of caregiver.

It is however recommended that Government and other relevant stakeholders need to institute continuous awareness campaign programmes on malnutrition and its adverse effects on primary school pupils learning and future attainments. They should also further improve and strengthen the training of caregivers on malnutrition to better improve the presently achieved good knowledge rate and to ensure implementation of this knowledge in the nutrition of their children in order to reduce malnutrition.

## Limitations

This study did not assess the dietary intake of the school children, therefore, it could not be concluded if the poor nutritional status found by this study is also associated with dietary intake aside other reported factors.

## Supporting information

S1 File (XLSX)
