# Correction: Nutritional status of primary school children and their caregiver’s knowledge on malnutrition in rural and urban communities of Ekiti State, Southwest Nigeria

**DOI:** 10.1371/journal.pone.0309680

**Published:** 2024-08-27

**Authors:** Taofeek Adedayo Sanni, Olusegun Elijah Elegbede, Kayode Rasak Adewoye, Kabir Adekunle Durowade, Tope Michael Ipinnimo, Ayodele Kamal Alabi, John Olujide Ojo, Richard Dele Agbana, Mustapha Muhammad Raji, Oluseyi Adedeji Aderinwale, Mojoyinola Oyindamola Adeosun, Ademuyiwa Adetona, Opeyemi Oladipupo Abioye, Olumide Temitope Asake, Olanrewaju Kassim Olasehinde, Olawale Bashir-ud-deen Oni

The affiliation for the fifth author is incorrect. Tope Michael Ipinnimo is not affiliated with #2 but with #1: Department of Community Medicine, Federal Teaching Hospital, Ido-Ekiti, Ekiti State, Nigeria.
